# Awns Enhance the Thousand Seed Weight of *Elymus nutans* Griseb. by Regulating Carbohydrate Contents and Gene Expression

**DOI:** 10.3390/biology15110874

**Published:** 2026-06-01

**Authors:** Yongsen Qiu, Huanhuan Lu, Yancui Zhao, Liuban Tang, Fei Zhang, Rui Zhang, Wengang Xie

**Affiliations:** State Key Laboratory of Herbage Improvement and Grassland Agro-Ecosystems, Key Laboratory of Grass-Land Livestock Industry Innovation, Ministry of Agriculture, College of Pastoral Agriculture Science and Technology, Lanzhou University, Lanzhou 730020, China

**Keywords:** awns, *Elymus nutans* Griseb., thousand seed weight, soluble sugar, starch, ascorbate and aldarate metabolism

## Abstract

Awns are crucial spike traits in Poaceae plants and are closely associated with seed development. *Elymus nutans* Griseb. is a high-quality alpine forage and an essential grass species for ecological restoration. To reveal the regulatory mechanism of awns on thousand seed weight in *E. nutans*, 20 *E. nutans* germplasm accessions were used as experimental materials in this study. Superior germplasms were screened via phenotypic correlation analysis. The screened superior germplasm was subjected to awned and de-awned treatments. Physiological indicators during seed development under the two treatments were measured at the milk stage, dough stage, and full ripe stage, and transcriptome sequencing was further used to identify the core regulatory pathways and key genes. The results showed that awn length was extremely significantly positively correlated with thousand seed weight (*p* < 0.01), and the optimal germplasm PI 655186 with superior awn length and thousand seed weight was obtained. Compared with the de-awned treatment, the awn-retained treatment significantly increased the contents of soluble sugar and starch in seeds from the milk stage to the dough stage and enhanced the activities of SOD, CAT, and POD to maintain redox homeostasis. Transcriptome analysis indicated that differentially expressed genes were significantly enriched in pathways including starch and sucrose metabolism and ascorbate and aldarate metabolism, among which *UGDH*, *GLCAK*, *VTC2_5*, and *APX* were identified as key genes regulating seed development, and WGCNA showed that the brown module was significantly correlated with soluble sugars and starch, with hub genes consisting of seven 60S ribosomal proteins and one prolyl 4-hydroxylase. In conclusion, awns positively affect the thousand seed weight of *E. nutans* seeds by promoting the synthesis of storage substances, optimizing the antioxidant enzyme system, and regulating the ascorbate and aldarate metabolism pathway and the expression of hub genes. These findings clarify the physiological and molecular mechanisms by which awns regulate seed thousand seed weight and provide a theoretical basis and gene resources for the innovation of high-yield and high-quality germplasms of alpine forage grasses.

## Simple Summary

Awns are important spike traits of Poaceae plants, and *Elymus nutans* Griseb. is a high-quality alpine forage with great significance for ecological restoration. To explore how awns affect the thousand seed weight of *E. nutans*, we used 20 *E. nutans* germplasm accessions as materials, screened superior germplasm, and conducted awned and de-awned treatments. We measured physiological indicators during seed development and performed transcriptome sequencing. The results showed that awn length was significantly positively correlated with thousand seed weight, and the superior germplasm PI 655186 was screened out. Compared with de-awned treatment, awned treatment increased soluble sugar and starch contents in seeds and enhanced antioxidant enzyme activities. Transcriptome analysis identified key genes and metabolic pathways related to seed development. In conclusion, awns can promote thousand seed weight of *E. nutans* by regulating storage substance synthesis, antioxidant systems, and related genes. This study provides a theoretical basis for the innovation of high-yield and high-quality alpine forage germplasm.

## 1. Introduction

Awns are important structures in the spikes of gramineous crops and are products of long-term environmental adaptation and evolution in plants [1]. As important trait characteristics of seeds, the length and morphological types of awns serve as key morphological markers for distinguishing grain varieties [2,3]. The effects of awns on seeds run through the entire processes of seed development, germination, dispersal, and yield formation [4]. The core lies in their functions as photosynthetic organs to enhance grain-filling efficiency and improve stress resistance while optimizing seed burial and germination through physical structures; however, they may also cause yield fluctuations due to resource competition [5,6,7].

The effect of awns on seed yield is complex, and their effects vary with plant species and growth environments. Some studies have pointed out that the longer the awns in wheat (*Triticum aestivum* L.), the greater the contribution of spike photosynthesis to grain filling, especially under source-limited conditions such as defoliation and drought [8]. Rebetzke reported that awned near-isogenic wheat lines performed better under stressful environments, including high temperature, drought, and rain-fed conditions [9]. Under drought stress, de-awned treatment could reduce spike photosynthesis and grain yield in wheat [7]. Similarly, Olugbemi and Motzo [10,11] found that awned wheat yielded higher than awnless wheat under drought conditions, whereas this advantage was absent under irrigated conditions. However, Sanchez-Bragado observed no significant yield differences between awned and awnless wheat, and in most cases, the yield gain or loss caused by the presence of awns was negligible [12,13]. During the grain-filling stage of barley (*Hordeum vulgare* L.), awns act as important photosynthetic organs, and awn-clipping treatment exerts significant effects on grain weight for awned germplasm [6]. Barley awns play an important positive role in yield and water use efficiency under drought conditions [14]. Nevertheless, a study by Hosseini [15] showed no significant correlation between the total photosynthesis of awns and yield for some tested germplasm under both drought and irrigated conditions, and a larger awn area in barley did not result in higher yield. Additionally, not all awns possess photosynthetic capabilities. For instance, studies on the Andropogoneae (a tribe in the grass subfamily Panicoideae) have detected no carbon assimilation activity in the awns attached to seed bearing spikelets [16,17]. The growth and persistence of awns impose a resource cost [18]. It has been proposed that awn development reflects an adaptive shift in reproductive strategy, moving from the production of many small seeds to fewer but nutritionally more substantial seeds [4].

During seed maturation, carbohydrate contents present a dynamic pattern in which soluble sugar decreases continuously, while starch accumulates progressively and tends to be stable. At the milk stage, grain filling begins, and abundant soluble sugars (mainly sucrose) are transported into the endosperm, initiating and promoting a rapid increase in starch synthesis [19]. During this period, the synthesis of macromolecular metabolites is the most vigorous in rice (*Oryza sativa* L.) grains, with massive accumulation of starch and amylose [20]. At the dough stage, starch synthesis reaches its peak, soluble sugar content declines markedly, and dry matter accumulation approaches the maximum level [21]. For example, the concentrations of soluble sugar and sucrose in wheat grains peak at 7 days after flowering and then decrease continuously, while starch synthesis increases rapidly from the 14th day after flowering [22]. At the full ripe stage, starch content remains stably at a high level, soluble sugar content drops to the lowest value, seeds complete dehydration and drying, and carbohydrate metabolism basically ceases [23]. The contents of soluble sugar and sucrose in different components of *Elymus sibiricus* L. seeds decrease significantly from the milk stage to the dough stage (*p* < 0.05) [24]. Overall, crops such as rice, wheat and *E. sibiricus* realize the efficient conversion of soluble sugars to storage starch from the milk stage to the full ripe stage, which reserves energy for seed germination.

The genus *Elymus* is the largest genus in the tribe Triticeae of the family Poaceae, comprising more than 150 species that are closely related to major cereal crops such as wheat, barley, and rye (*Secale cereale* L.) [25,26]. These wild *Elymus* species are widely used in ecological restoration, forage breeding, and cereal crop improvement [27,28]. *E. nutans* is a perennial, self-compatible, long-awned allohexaploid herb (2n = 6x = 42, genome composition StStYYHH) that is widely distributed on the Qinghai–Tibet Plateau [29,30]. This species not only exhibits excellent forage traits but also possesses strong tolerance to multiple abiotic stresses, including drought, low temperature, and radiation, stress resistances that are generally lacking in cereal crops [31,32,33,34]. The yield of *E. nutans* (including seed yield and biomass yield) is constrained by factors such as agronomic traits, water and fertilizer management, and environmental conditions, which pose significant challenges to its genetic improvement and breeding programs [35,36,37,38].

Crop yield is determined by the number of grains per spike and the average grain weight. Multiple independent studies have reported that the presence of awns leads to larger and heavier grains across various genetic backgrounds [14,39,40,41,42]. Our previous studies have demonstrated that awns of *Elymus nutans* exert positive effects on seed yield per plant and thousand seed weight [43]. However, the physiological and molecular mechanisms by which awns affect the thousand seed weight of *E. nutans* remain unclear. Based on this, the present study investigated the physiological and molecular mechanisms underlying the effect of awns on thousand seed weight of *E. nutans* seeds under de-awned treatment, aiming to provide a theoretical reference for improving spike-related traits and breeding high-yield, stress-tolerant varieties in gramineous crops.

## 2. Materials and Methods

### 2.1. Experimental Materials, Treatments, and Growth Conditions

The experiment was conducted using 20 accessions of *E. nutans* as materials (Table 1). On 28 April 2023, healthy and plump seeds were sown in pots in the greenhouse of Lanzhou University, Yuzhong Campus, Lanzhou, Gansu Province. Each accession was planted in 5 pots, with 4 seeds per pot. After 28 days of cultivation, 15 healthy and uniformly growing seedlings from each accession were transplanted into the germplasm nursery. The experiment was arranged in a randomized complete block design (RCBD) with three biological replicates. Each plot served as the basic experimental unit and contained five individual plants. Each plot was 2.5 m in length and 0.6 m in width, with an area of 1.5 m^2^. A total of 60 plots were randomly arranged within each block.

In 2023, we evaluated awn length and thousand seed weight of 20 *E. nutans* accessions at the full ripe stage in late August. To explore the effect of awn length on thousand seed weight, the material with the longest awn length (PI 655186) was selected in 2024, and awning removal treatment was performed at the heading stage on 4 July 2024. Three individual plants with consistent growth vigor were selected for treatment. For de-awning treatment, the awns of seeds on half of each spike were removed with scissors, while the other half were retained as the control. Based on the treated experimental materials, physiological index determination and next-generation transcriptome sequencing were performed on seeds of the two groups at the milk stage (1 August 2024), dough stage (8 August 2024), and full ripe stage (15 August 2024). Three biological replicates were collected at each stage. All samples were immediately frozen in liquid nitrogen and stored at −80 °C. Both physiological measurement and transcriptome sequencing adopted the same batch of samples.

The experimental site is located at 35°56′57″ N, 104°9′13″ E, with an altitude of 1720 m. In 2023, the annual average temperature was 8.1°C and the total annual precipitation was 320.5 mm. In 2024, the annual average temperature was 8.5°C, and the total annual precipitation was 421.4 mm. This area features a typical temperate semi-arid continental monsoon climate. The soil in the experimental plot is yellow loam with an organic matter content of 2.4% and a pH value of 7.6.

### 2.2. Determination of Phenotypic and Physiological Indexes

Awn length of each germplasm was measured using a vernier caliper with 8 replicates. Thousand seed weight was determined by the hundred-seed method via random weighing for each germplasm, with 8 replicates. Physiological indices of seeds from the two treatment groups of PI 655186 were determined at the milk stage, dough stage, and full ripe stage. Three biological replicates were sampled at each stage. Commercially available kits purchased from Shanghai Enzyme-linked Biotechnology Co., Ltd., Shanghai, China. were used to determine the activities of superoxide dismutase (SOD), catalase (CAT), and peroxidase (POD), as well as the contents of soluble sugar, starch, crude fat, and soluble protein in samples. All indexes were set with 3 replicates.

### 2.3. Transcriptome Sequencing

#### 2.3.1. RNA Extraction, Library Construction, Sequencing, and Gene Annotation

Total plant RNA was extracted using the RNA prep Pure Plant Kit (Tiangen, Beijing, China). RNA integrity was evaluated using the RNA Nano 6000 Assay Kit (Agilent Technologies, Santa Clara, CA, USA) with the Agilent Bioanalyzer 2100 system (Agilent Technologies, Santa Clara, CA, USA). A cDNA library was constructed for each sample, with 18 cDNA libraries established in total. After passing quality inspection, the libraries were subjected to high-throughput transcriptome sequencing using the Illumina NovaSeq 6000 platform (Illumina Inc., San Diego, CA, USA). Clean sequencing data were obtained after quality control using Fastp v1.3.3 to remove redundant sequences, with Q30 and GC content calculated. The clean reads were aligned to the reference genome (https://ngdc.cncb.ac.cn/gwh/Assembly/85969/show) (accessed on 12 September 2025) using HISAT2 v2.2.1 to obtain gene annotations. Gene expression levels were quantified by RSEM v1.2.19 with the FPKM method. The transcriptome sequencing was performed by Biomarker Technologies Co., Ltd., Beijing, China.

#### 2.3.2. Identification and Enrichment Analysis of Differentially Expressed Genes (DEGs)

To explore differences in gene expression levels between different treatment groups, principal component analysis (PCA) was performed using the “FactoMineR” package in R (version 4.4.2), and PCA scatter plots were generated with the “ggplot2” package. Comparisons were set as WA vs. YA, WB vs. YB, and WC vs. YC, where W denotes de-awned plants, Y denotes awned plants, and A, B, and C represent the milk stage, dough stage, and full ripe stage, respectively. Upregulation indicates higher expression in awned samples. Differentially expressed genes (DEGs) were identified using DESeq2 v1.42.0 with a fold change (FC) ≥ 1.5 and a false discovery rate (FDR) < 0.01. Based on the reference genome, GO terms and KEGG pathways were enriched using the GO Enrichment and KEGG Enrichment Analysis plugins in TBtools v2.376 and visualized via OmicShare Tools (https://www.omicshare.com/tools) (accessed on 10 November 2025).

#### 2.3.3. Weighted Gene Co-Expression Network Analysis (WGCNA)

Weighted gene co-expression network analysis (WGCNA) was performed using the R (version 4.4.2) package “WGCNA” [44]. To obtain candidate genes related to the effects of awns on seed thousand seed weight, weighted gene co-expression network analysis (WGCNA) was conducted using all differentially expressed genes and seven seed physiological indicators. The thresholds were set as follows: gene expression level of 1.5, module similarity of 0.25, and minimum gene number within each module of 30. Physiological data collected at the same sampling stages as transcriptome samples were adopted for WGCNA analysis. To identify core genes regulating thousand seed weight under awn treatment, hub gene co-expression networks were screened using the MCODE plugin in Cytoscape v3.10.4 based on the highest Degree scores. Module membership (kME) was defined as the Pearson correlation coefficient between each gene’s expression profile and the module eigengene (the first principal component of the module). Gene significance (GS) was defined as the absolute value of the Pearson correlation between gene expression and the trait of interest, representing the strength of association.

#### 2.3.4. Quantitative Real-Time PCR (qRT-PCR) Validation

According to transcriptome analysis results, 12 DEGs were selected for qPCR validation. The RNA used for detection was the same as that used for transcriptome analysis. Gene-specific primers were designed using Primer 6.0 software (Table 2). *E. nutans* glyceraldehyde-3-phosphate dehydrogenase (*EnGAPDH*) was used as the internal reference gene [45]. Reagents for qRT-PCR quantification included AG RNAex Pro RNA Extraction Reagent (AG21101), Evo M-MLV Reverse Transcriptase Premix (AG11706) for reverse transcription, and SYBR Green Pro Taq HS Premix qPCR Kit II (AG11718). All these reagents were purchased from Hunan Accurate Biotechnology Co., Ltd., Changsha, China. The relative expression levels of genes were calculated using the 2^−ΔΔCt^ method [46].

### 2.4. Data Analysis

Data were organized using Excel 2021. To investigate phenotypic differences among germplasms, trait correlations, and the effect of awns on thousand seed weight, one-way analysis of variance (ANOVA) with Duncan’s multiple range test, Pearson correlation analysis, and linear regression analysis were performed using SPSS v26.0, based on awn length and thousand seed weight data from 20 *E. nutans* germplasm accessions. For thousand seed weight and physiological indicators of PI 655186, differential analysis with paired-sample *t*-tests and visualization were conducted for each indicator via the online tool Chiplot (https://www.chiplot.online) (accessed on 15 January 2026). Pearson correlation analysis was used to verify the correlation between RNA-seq and qRT-PCR expression values. * indicates significance at *p* < 0.05; ** indicates significance at *p* < 0.01.

## 3. Results

### 3.1. Determination of Phenotypic Data and Material Screening

**Figure 1:**
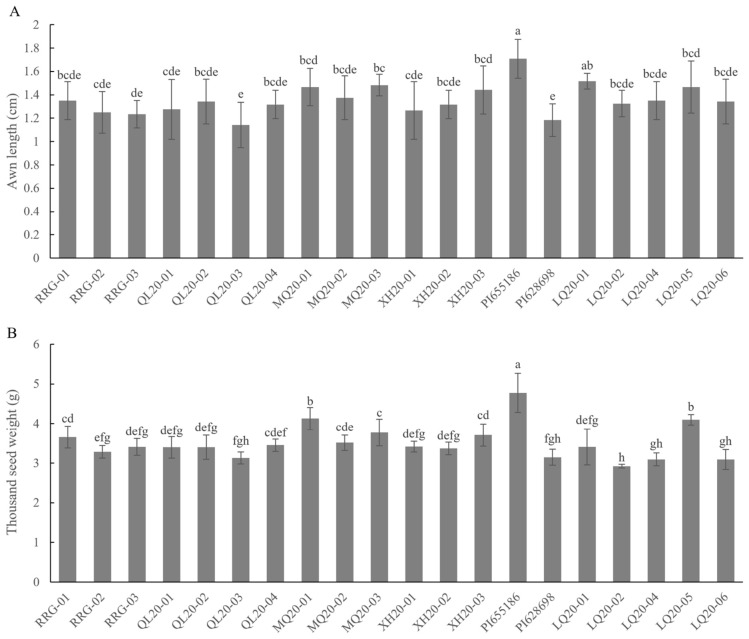
Awn length and thousand seed weight of 20 *E. nutans* accessions. (**A**) Awn length; (**B**) Thousand seed weight. Different lowercase letters (a–h) above the bars indicate significant differences (*p* < 0.05) among accessions according to Duncan’s multiple range test.

**Figure 2:**
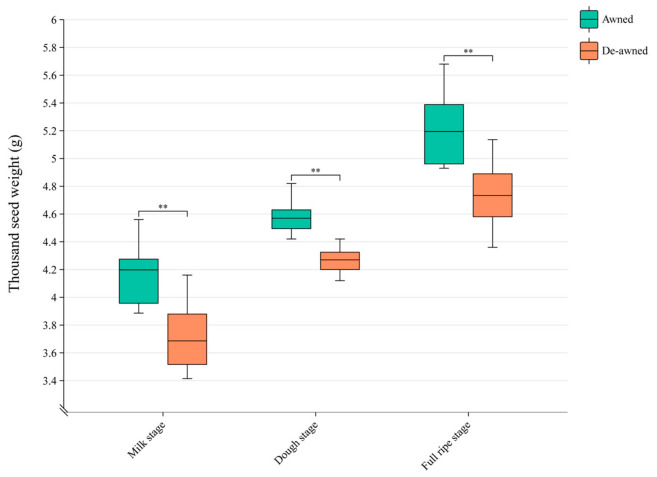
Thousand seed weight of PI 655186 at three stages under two treatments. *, ** denote differences between treatments at *p* < 0.05 and *p* < 0.01, respectively.

Awn length and thousand seed weight of the 20 *E. nutans* accessions are shown in Figure 1. Awn length exhibited an extremely significantly positive correlation with thousand seed weight (r = 0.794; *p* < 0.01). Regression analysis with awn length as the independent variable and thousand seed weight as the dependent variable indicated a highly significant positive linear relationship of awn length on thousand seed weight (*p* < 0.01), with a coefficient of determination R^2^ = 0.63 and the regression equation TSW = −0.096 + 2.655 × Awn. The results demonstrated that awns exert a positive effect on thousand seed weight of *E. nutans* seeds. The accession PI 655186, with the longest awns and the highest thousand seed weight (awn length: 1.708 cm; thousand seed weight: 4.777 g), was selected for subsequent research. At all three stages, the thousand seed weight of PI 655186 under the awned treatment was significantly higher than that under the de-awned treatment (*p* < 0.01) (Figure 2).

### 3.2. Analysis of Physiological Indexes of PI 655186

#### 3.2.1. Analysis of Nutrient and Storage Substance Contents

**Figure 3:**
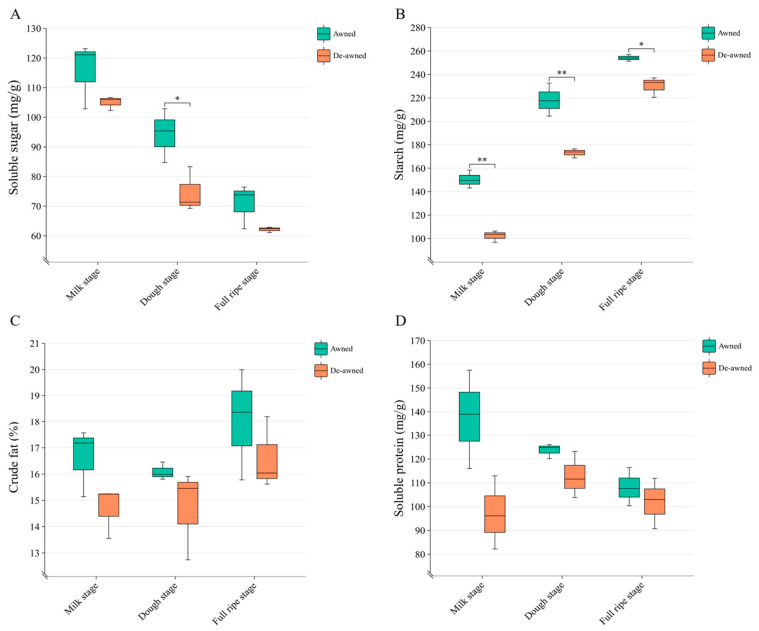
Changes in storage substances of PI 655186 at different developmental stages. (**A**) Soluble sugar; (**B**) Starch; (**C**) Crude fat; (**D**) Soluble protein. *, ** denote differences between treatments at *p* < 0.05 and *p* < 0.01, respectively.

At different growth stages, the contents of soluble sugar, starch, crude fat, and soluble protein in seeds differed between the two treatments (Figure 3). The soluble sugar content showed a continuous decreasing trend with seed development. At the dough stage, the soluble sugar content under the awned treatment was 26.36% higher than that under the de-awned treatment (*p* < 0.05). No significant differences were observed between the two treatments at the milk stage and full ripe stage. The starch content showed a continuous increasing trend with seed development. At the milk, dough, and full ripe stages, the starch content under the awned treatment was 47.11%, 26.07%, and 10.39% higher than that under the de-awned treatment, respectively (*p* < 0.05; *p* < 0.01). The crude fat content and soluble protein content under the awned treatment were higher than those under the de-awned treatment at all three stages, but the differences were not significant.

#### 3.2.2. Analysis of Antioxidant Enzyme Activities

**Figure 4:**
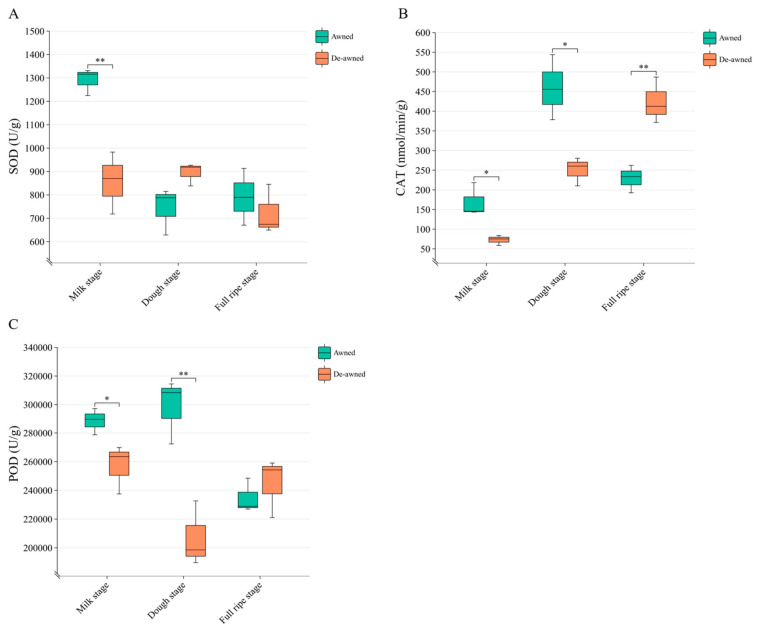
Changes in antioxidant enzyme activities of PI 655186 at different developmental stages. (**A**) SOD; (**B**) CAT; (**C**) POD. *, ** denote differences between treatments at *p* < 0.05 and *p* < 0.01, respectively.

Changes in seed antioxidant enzyme activities at different growth stages are shown in Figure 4. At the milk stage, SOD, CAT, and POD activities under the awned treatment were 50.57%, 132.57%, and 12.3% higher than those under the de-awned treatment, respectively (*p* < 0.05; *p* < 0.01). At the dough stage, CAT and POD activities under the awned treatment were 83.54% and 44.2% higher than those under the de-awned treatment, respectively (*p* < 0.05; *p* < 0.01), whereas no significant difference in SOD activity was observed between the two treatments. At the full ripe stage, CAT activity under the de-awned treatment was 84.65% higher than that under the awned treatment (*p* < 0.01), and no significant differences in SOD and POD activities were found between the two treatments. Overall, the peak antioxidant enzyme activities under the awned treatment were concentrated from the milk stage to the dough stage, whereas increased enzyme activities under the de-awned treatment mainly occurred from the dough stage to the full ripe stage.

### 3.3. Screening of Differentially Expressed Genes

**Figure 5:**
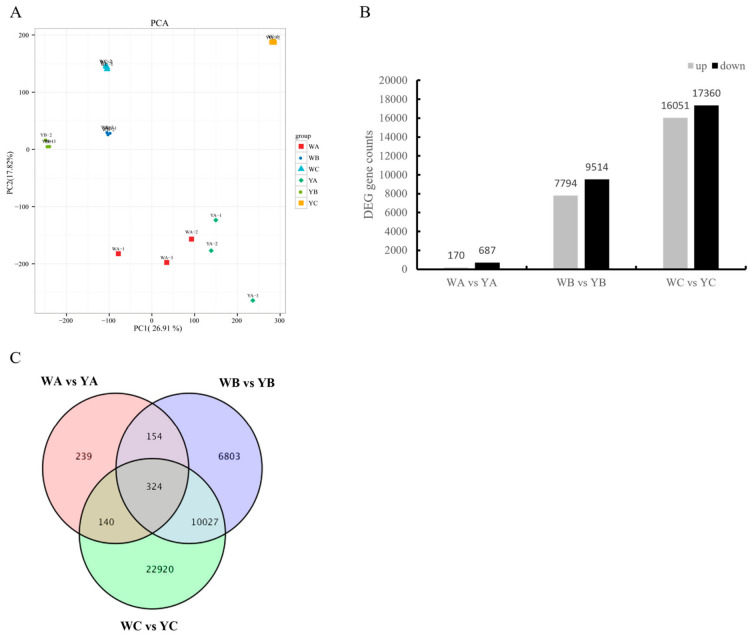
Principal component analysis of samples and number of differentially expressed genes. (**A**) Principal component analysis chart; (**B**) Bar chart; (**C**) Venn diagram. Comparisons were set as WA vs. YA, WB vs. YB, and WC vs. YC, where W denotes de-awned plants, Y denotes awned plants, and A, B, and C represent the milk stage, dough stage, and full ripe stage, respectively.

To further elucidate the molecular mechanism underlying the effect of awns on thousand seed weight of *E. nutans* seeds, transcriptome analysis was performed on the awned treatment group (Y-) and de-awned treatment group (W-) at the milk stage (-A), dough stage (-B), and full ripe stage (-C). A total of 18 samples were subjected to transcriptome analysis. After sequencing quality control, 122.54 Gb of Clean Data were obtained, and the percentage of Q30 bases in each sample was no less than 91.32% (Table A1). The clean reads of each sample were sequentially mapped to the designated reference genome, with mapping efficiencies ranging from 69.80% to 85.52%, which met the requirements for subsequent analysis. For non-model species, the reference genome or transcriptome is often characterized by incomplete assembly and poor annotation, which directly results in a proportion of reads failing to map effectively. The evolutionary distance between the reference species and the sequenced species can also influence mapping rates [47]. The principal component analysis plot of samples is shown in Figure 5A. In this study, genes with Fold Change (FC) ≥ 1.5 and False discovery rate (FDR) < 0.01 were set as the screening thresholds for differentially expressed genes (DEGs). As shown in Figure 5B, a total of 857 DEGs (170 upregulated and 687 downregulated) were identified in the WA vs. YA comparison group; 17,308 DEGs (7794 upregulated and 9514 downregulated) were identified in the WB vs. YB comparison group; and 33,411 DEGs (16,051 upregulated and 17,360 downregulated) were identified in the WC vs. YC comparison group. Venn diagram analysis of DEGs among different comparison combinations revealed a total of 324 common DEGs (Figure 5C).

### 3.4. GO and KEGG Enrichment Analysis of Differentially Expressed Genes

**Figure 6:**
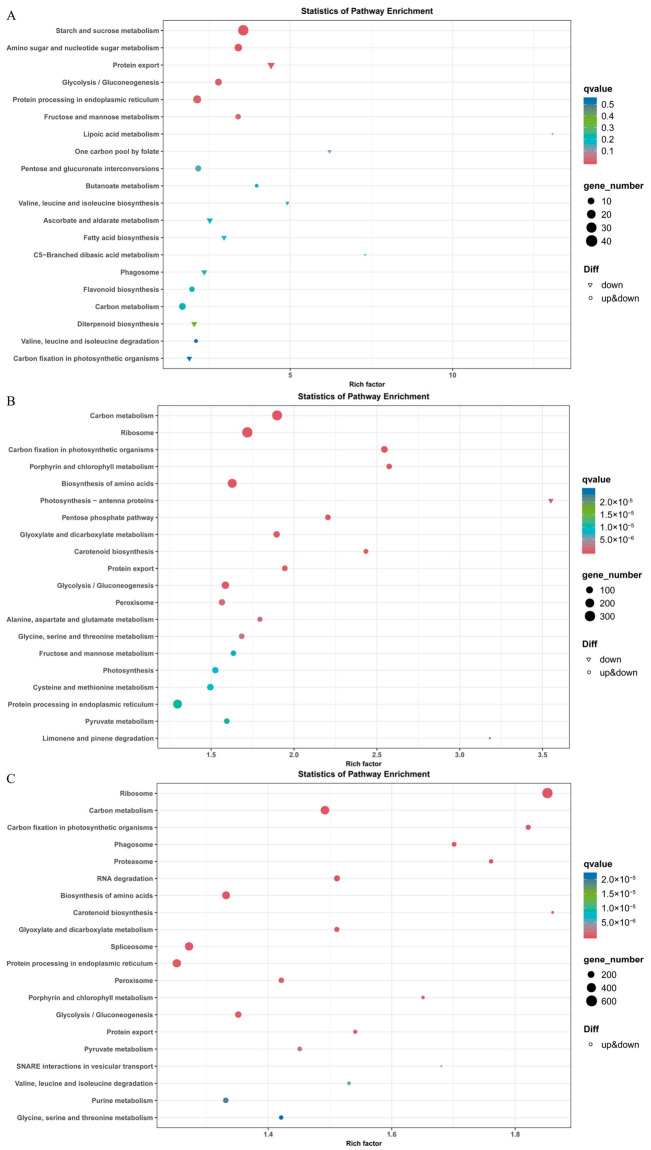
KEGG pathway enrichment analysis of DEGs. (**A**) WA vs. YA; (**B**) WB vs. YB; (**C**) WC vs. YC. Comparisons were set as WA vs. YA, WB vs. YB, and WC vs. YC, where W denotes de-awned plants, Y denotes awned plants, and A, B, and C represent the milk stage, dough stage, and full ripe stage, respectively.

**Figure A1:**
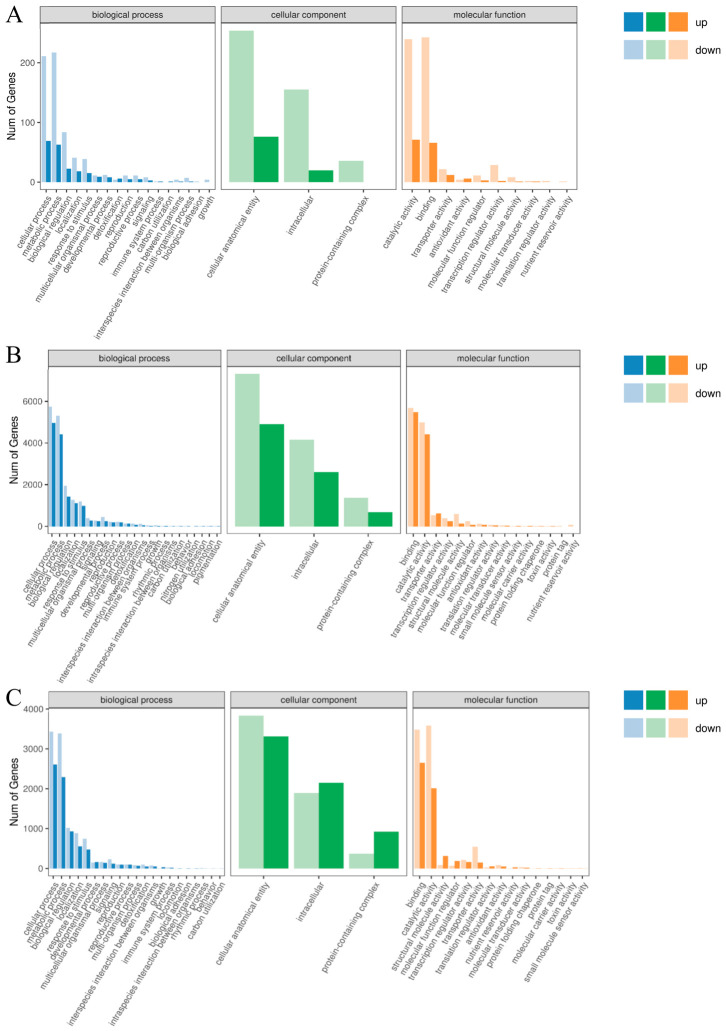
Statistical Chart of GO Annotation Classification for Differentially Expressed Genes. (**A**) WA vs. YA; (**B**) WB vs. YB; (**C**) WC vs. YC.

GO and KEGG enrichment analyses were performed based on the DEGs from each comparison group. DEGs were mainly categorized into three major groups: Biological Process (BP), Cellular Component (CC), and Molecular Function (MF), and their distribution in the GO hierarchy is shown in Figure A1. Among the three comparison groups, DEGs enriched in the BP category were mainly annotated to terms such as cellular process, metabolic process, and biological regulation; those in the CC category were significantly enriched in cellular anatomical entity, intracellular, and protein-containing complex; and those in the MF category were mainly enriched in binding, catalytic activity, and transporter activity. The top 20 KEGG enrichment pathways of DEGs at the three developmental stages are presented in Figure 6, with distinct pathways enriched across the comparison groups. The top five significantly enriched pathways in the WA vs. YA comparison group included: Starch and sucrose metabolism, Amino sugar and nucleotide sugar metabolism, Glycolysis/Gluconeogenesis, Protein processing in endoplasmic reticulum, and Fructose and mannose metabolism (Figure 6A). For the WB vs. YB comparison group, the top five significantly enriched pathways were: Carbon metabolism, Ribosome, Biosynthesis of amino acids, Carbon fixation in photosynthetic organisms, and Porphyrin and chlorophyll metabolism (Figure 6B). In the WC vs. YC comparison group, the top five significantly enriched pathways were: Ribosome, Carbon metabolism, Carbon fixation in photosynthetic organisms, Biosynthesis of amino acids, and Glycolysis/Gluconeogenesis (Figure 6C). The enriched pathways in the dough stage comparison group (WB vs. YB) and full ripe stage comparison group (WC vs. YC) exhibited high similarity. Notably, DEGs in the WA vs. YA comparison group were significantly enriched in the Ascorbate and aldarate metabolism pathway, and all DEGs were downregulated. This pathway was also significantly enriched in WB vs. YB and WC vs. YC, with DEGs showing both upregulated and downregulated expression patterns.

### 3.5. Analysis of Ascorbate and Aldarate Metabolism Pathway

**Figure 7:**
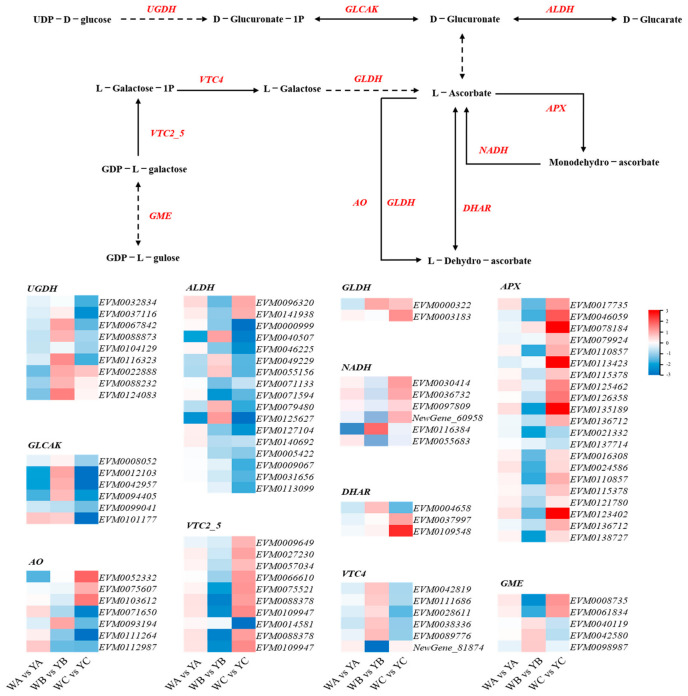
Putative ascorbate and aldarate metabolism network in *E. nutans.* Comparisons were set as WA vs. YA, WB vs. YB, and WC vs. YC, where W denotes de-awned plants, Y denotes awned plants, and A, B, and C represent the milk stage, dough stage, and full ripe stage, respectively.

The above analysis revealed that the ascorbate and aldarate metabolism pathway plays a key role in determining the effect of awns on seed thousand seed weight. Therefore, in-depth exploration was conducted on its key regulatory mechanism, and the ascorbate and aldarate metabolism network of *E. nutans* was deduced (Figure 7). A total of 92 DEGs involved in the ascorbate and aldarate metabolism pathway were identified, covering 11 genes, specifically: *UGDH* (UDP-glucose 6-dehydrogenase) with 9 DEGs, *GLCAK* (glucuronokinase) with 6 DEGs, *ALDH* (aldehyde dehydrogenase) with 17 DEGs, *NADH* (monodehydroascorbate reductase) with 6 DEGs, *VTC2_5* (GDP-L-galactose phosphorylase) with 10 DEGs, *VTC4* (inositol-phosphate phosphatase) with 6 DEGs, *GLDH* (L-galactono-1,4-lactone dehydrogenase) with 2 DEGs, *GME* (GDP-D-mannose 3′,5′-epimerase) with 5 DEGs, *APX* (L-ascorbate peroxidase) with 21 DEGs, *DHAR* (glutathione dehydrogenase) with 3 DEGs, and *AO* (L-ascorbate oxidase) with 7 DEGs. At the milk stage, most DEGs of *VTC2_5* were upregulated, whereas most DEGs of *UGDH* and *GLCAK* were downregulated; at the dough stage, most DEGs of *UGDH*, *GLCAK*, and *VTC4* were upregulated, while most DEGs of *NADH*, *VTC2_5*, and *APX* were downregulated, among which DEGs of *VTC2_5* showed a deeper color (larger fold change); at the full ripe stage, most DEGs of *VTC2_5* and *APX* were upregulated, whereas most DEGs of *ALDH*, *GLCAK*, and *VTC4* were downregulated, among which DEGs of *GLCAK*, *ALDH*, and *APX* showed a deeper color (larger fold change).

### 3.6. Co-Expression Network of Soluble Sugar- and Starch-Related Regulatory Genes

**Figure 8:**
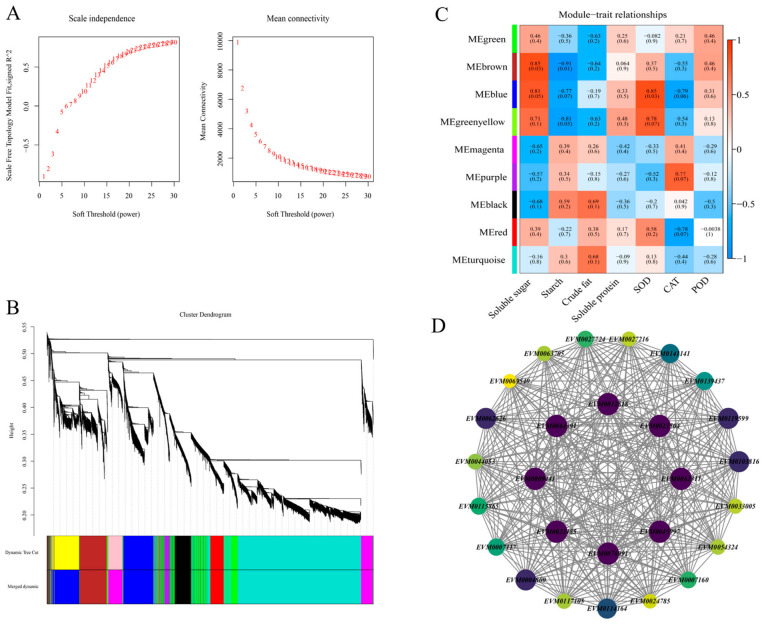
Weighted gene co-expression network analysis (WGCNA). (**A**) Scale-free soft threshold distribution diagram; (**B**) WGCNA gene clustering tree; (**C**) Correlation heatmap between WGCNA modules and traits; (**D**) Co-expression network analysis of brown modules.

The expression threshold was set to 1.5, the module similarity threshold to 0.25, and the minimum number of genes within a module to 30 (Figure 8A,B). A total of 9 gene expression modules were finally identified, which were associated with the indices of soluble sugar, starch, crude fat, soluble protein, SOD, CAT, and POD, respectively. Among them, the brown module was significantly positively correlated with soluble sugar (r = 0.85, *p* = 0.03) and significantly negatively correlated with starch (r = −0.91, *p* = 0.01) (Figure 8C). A total of 27 hub genes with scores greater than 26 were identified using MCODE and visualized (Figure 8D). A darker circle color indicates a higher involvement of the gene in regulating starch content. The top 8 hub genes with scores greater than 26.5 were annotated, and the annotation results revealed 7 60S ribosomal proteins and 1 prolyl 4-hydroxylase (Table 3).

### 3.7. qRT-PCR Validation

**Figure 9:**
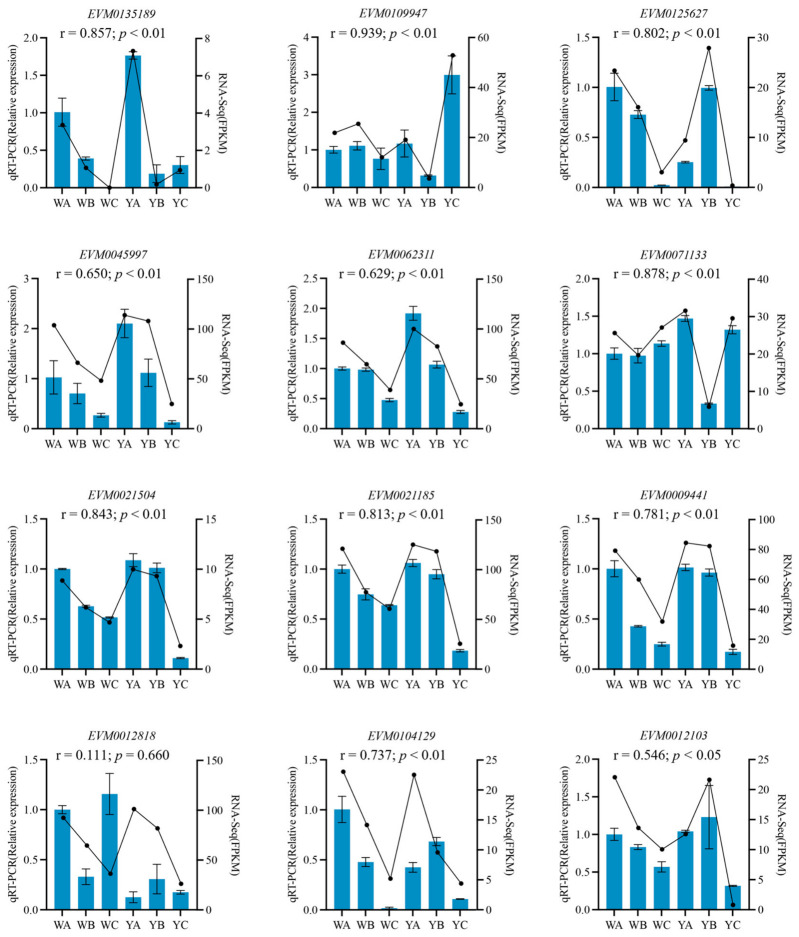
The expression levels of 12 differentially expressed genes were verified by qRTPCR.

The DEGs identified from transcriptome sequencing were validated by qRT-PCR. According to the annotation results, 12 genes were selected from WGCNA hub genes, the ascorbate and aldarate metabolism pathway, and other pathways for experimental verification (Figure 9). The results revealed that the expression levels of 11 genes showed a significant positive correlation between transcriptome sequencing and qRT-PCR (*p* < 0.05; *p* < 0.01). However, no significant correlation was observed between qRT-PCR results and transcriptome data for gene *EVM0012818* (*EnRPL26A*). In general, the expression trends of the 12 differentially expressed genes validated by qRT-PCR were basically consistent with the changes in FPKM values from transcriptome sequencing, indicating that the transcriptome sequencing data are highly reliable and can accurately reflect changes in gene expression levels.

## 4. Discussion

### 4.1. Relationship Between Awns and Seed Thousand Seed Weight in E. nutans

Studies on the phenotypic evolution of Triticeae grasses have shown that awns can significantly improve grain weight by participating in the synthesis and distribution of assimilates [48,49]. At present, studies on the effect of awns on seed yield of *Elymus species* are still relatively limited. Ntakirutimana investigated the effects of awns on seed yield-related traits in *E. sibiricus* and found that de-awned treatment reduced thousand seed weight and grain size under both irrigation and rainfall conditions, ultimately leading to decreased seed yield per plant [50]. Research by Qiu indicated that awns in *E. nutans* were significantly correlated with thousand seed weight and seed yield per plant [43]. In the present study, analysis of 20 *E. nutans* accessions revealed an extremely significant positive correlation between awn length and thousand seed weight (*p* < 0.01), and regression analysis also confirmed an extremely significant positive linear regulatory effect of awn length on thousand seed weight. These results are consistent with previous findings, suggesting that awns in *E. nutans* exert a positive effect on seed thousand seed weight.

### 4.2. Changes in Storage Substances of E. nutans Seeds Under Two Treatments

Seed development involves a series of complex and dynamic metabolic processes, including cell division and differentiation, as well as the biosynthesis of carbohydrates, proteins, lipids, amino acids, hormones, and secondary metabolites [51]. Starch is the main storage substance during seed development, and sucrose is one of the primary energy sources. Hydrolysis of sucrose affects starch synthesis, which in turn influences seed filling and grain size [19,52]. This study found that awn retention significantly increased the contents of soluble sugars and starch during seed development in *E. nutans*. In wheat, awns play a dominant role in carbohydrate production and grain weight contribution during the grain-filling stage [53]. Meanwhile, the accumulation of soluble sugars (especially sucrose and raffinose) during seed maturation and desiccation is closely associated with the acquisition of seed vigor [54]. Furthermore, studies on *Argania spinosa* have further demonstrated that the accumulation of soluble carbohydrates is a key metabolic adaptation mechanism ensuring seed formation under salt stress [55]. Several studies have reported the synthesis of storage substances during seed development in *Arabidopsis thaliana* [51,56]. In *Arabidopsis thaliana*, starch accumulates during the morphogenesis stage of seed development and then decreases, a process synchronized with sucrose accumulation at later developmental stages. Previous studies found that during *E. sibiricus* seed development, soluble sugar content gradually decreased with seed maturation, whereas starch content gradually increased [57]. Another study also reported that starch content in *E. sibiricus* seeds increased continuously and significantly from 5 to 20 days after flowering [58]. The present study revealed that from the milk stage to the full ripe stage, soluble sugar content in *E. nutans* seeds gradually decreased while starch content gradually increased, which is consistent with the characteristics of seed development. Seeds from awned plants had significantly higher soluble sugar and starch contents than those from de-awned plants (*p* < 0.05), whereas no significant differences were observed in crude fat and soluble protein contents. These findings support the conclusion that awns positively regulate thousand seed weight by early activation and continuous strengthening of the seed carbohydrate metabolic network.

### 4.3. Changes in Antioxidant Enzyme Activities in E. nutans Seeds Under Two Treatments

Reactive oxygen species (ROS) are produced during the important process of seed development and maturation, and ROS can cause effects such as rupture and denaturation of cell membrane lipid structures [59]. Antioxidant enzymes are the main enzymes for scavenging ROS. SOD can effectively eliminate reactive oxygen free radicals, POD can effectively protect membrane structures, and CAT can alleviate the stress of hydrogen peroxide. Therefore, antioxidant enzymes play a role in maintaining cell integrity and improving seed desiccation tolerance during seed development [60,61,62]. The present study found that during the development of *E. nutans* seeds, the antioxidant enzyme activities in the awned and de-awned treatment groups showed obvious temporal differences: the peak activities of SOD, CAT, and POD in the awned group were concentrated from the milk stage to the dough stage, whereas the increased enzyme activities in the de-awned group mainly occurred from the dough stage to the full ripe stage. Among them, the activities of SOD, CAT, and POD in the awned treatment at the milk stage were significantly higher than those in the de-awned treatment (*p* < 0.05). During seed maturation and desiccation in wheat, soluble sugar accumulation and changes in antioxidant enzyme activities exhibit temporal correlation; they act synergistically to ensure seed desiccation tolerance and germination potential [54]. Similarly, transcriptomic and metabolomic analyses following grain filling in rice show that antioxidant-related genes are highly coupled with carbon metabolic pathways such as glycolysis and gluconeogenesis in co-expression networks, indicating a coordinated regulatory relationship between the antioxidant system and carbohydrate metabolism during grain maturation [63]. In addition, under high-temperature stress in rice, salicylic-acid-induced soluble sugar accumulation, together with increased activities of antioxidant enzymes including SOD, POD, CAT, and APX, effectively protects floret development, further confirming the synergistic protective mechanism of carbohydrate signaling and the antioxidant enzyme system during reproductive development in gramineous crops [64]. Evidence from closely related gramineous species indicates that the coordinated regulation of antioxidant defense and carbohydrate metabolism during seed development is not incidental, but an important strategy for gramineous seeds to ensure grain-filling efficiency. In the present study, the temporal and spatial regulation of antioxidant enzyme activities by awns enables precise coupling of carbon metabolism and redox defense in seeds during the critical grain-filling period.

### 4.4. Ascorbate and Aldarate Metabolism Pathway as the Core Pathway for Awn-Regulated Seed Thousand Seed Weight

Ascorbic acid (AsA), also known as vitamin C, is one of the most important antioxidants in plants [65]. It scavenges reactive oxygen species through the redox cycle, and its metabolism and content changes directly affect plant growth and developmental processes such as flowering regulation, seed maturation, and senescence [66]. Ascorbate and aldarate metabolism belongs to carbohydrate metabolism in a broad sense. In the commonly used KEGG database for bioinformatics, it is clearly classified as a sub-pathway of Carbohydrate metabolism. During artificial aging and priming of wheat seeds, ascorbate and aldarate metabolism changes coordinately with several other Carbohydrate metabolism pathways (glycolysis, TCA cycle, starch and sucrose metabolism) [67]. In the present study, KEGG enrichment analysis revealed that the ascorbate and aldarate metabolism pathway was significantly enriched at all three growth stages. Further systematic analysis of this pathway identified a total of 92 differentially expressed genes involving 11 key genes, among which *UGDH*, *GLCAK*, *VTC2_5*, and *APX* were recognized as the core genes for awns regulating thousand seed weight in *E. nutans*. *UGDH* catalyzes the conversion of UDP-glucose to UDP-glucuronic acid (UDP-GlcA), serving as a key node linking primary carbon metabolism, cell wall polysaccharide synthesis, and ascorbate metabolism [68]. *GLCAK* catalyzes the phosphorylation of D-glucuronic acid (D-GlcA) to D-glucuronic acid-1-phosphate, which is subsequently converted to UDP-GlcA to replenish the precursor pool for cell wall and AsA synthesis, acting as a key branching node connecting sugar metabolism, cell wall synthesis, and AsA synthesis [69]. *VTC2/VTC5* encodes GDP-L-galactose phosphorylase, which catalyzes the first critical rate-limiting step in the main plant AsA biosynthetic pathway (Smirnoff–Wheeler pathway) and is the core enzyme regulating AsA biosynthesis in plants [70]. *APX* uses AsA as an electron donor to catalyze the reduction of hydrogen peroxide (H_2_O_2_) to water (H_2_O), representing the core enzyme for ROS scavenging and defense against oxidative stress in plants [71]. These four genes function synergistically in the ascorbate and aldarate metabolism pathway: *UGDH* and *GLCAK* are located upstream, responsible for supplying key precursors such as UDP-GlcA and linking starch and sucrose metabolism, aldarate metabolism, and AsA synthesis; *VTC2/VTC5* controls the metabolic flux of the main AsA biosynthetic pathway; and *APX* maintains AsA redox homeostasis, collectively ensuring the balance of carbon metabolism and redox homeostasis in plants.

### 4.5. WGCNA Screening of Hub Genes Related to Seed Storage Substance Accumulation

WGCNA results showed that the brown module was significantly positively correlated with soluble sugar content (*p* < 0.05) and significantly negatively correlated with starch content (*p* < 0.01). The brown module represents the core module underlying awn-regulated seed carbohydrate metabolism. Further analysis screened 8 hub genes, among which 7 were annotated as 60S ribosomal proteins and 1 was annotated as prolyl 4-hydroxylase. 60S ribosomal proteins are essential components of the large ribosomal subunit, whose core function is to control the overall intracellular protein synthesis rate by regulating translation elongation efficiency [72]. Starch synthesis is a complex process catalyzed by multiple enzymes, and the translation efficiency of these enzymes largely depends on normal ribosomal function [73]. In maize, the *DEK58* gene is involved in the assembly of the 60S ribosomal subunit. Loss of its function not only causes impaired ribosome assembly and blocked pre-rRNA processing but also leads to abnormal endosperm development and significantly reduced accumulation of storage reserves in grains [74]. Knockout mutation of *RPP1A* (encoding 60S ribosomal phosphoprotein P1A) in *Arabidopsis thaliana* resulted in a significantly decreased abundance of proteins involved in carboxylic acid metabolism and lipid biosynthesis in mature seeds, together with severe reductions in organic acids of the tricarboxylic acid cycle and carbohydrates of the pentose phosphate pathway [75]. Downregulated expression of specific 60S ribosomal protein genes directly affects cell division and seed morphology. RNAi silencing of *NtRPL17* (60S ribosomal protein L17) in tobacco leads to G2/M cell cycle arrest, reduced cell number, and ultimately markedly smaller embryos and seeds [76]. Carbohydrates such as sucrose can significantly induce the expression of numerous ribosomal protein genes, suggesting that carbon resource availability regulates the de novo synthesis of new ribosomes and thereby coordinates seed developmental progression [77]. Thus, normal expression of 60S ribosomal proteins is critical for maintaining the translation efficiency of starch-synthesizing enzymes and starch accumulation.

*P4H3* belongs to the prolyl 4-hydroxylase family and directly regulates the maturation and function of arabinogalactan proteins (AGPs) by hydroxylating the core proteins of cell wall glycoproteins, including AGPs. As key structural components of the cell wall, properly modified AGPs are essential for maintaining cell wall integrity, mechanical strength, and the stable structure of polysaccharide networks such as pectin [78]. In tomato, altered expression of *SlP4H3* not only affects the content and modification pattern of AGPs but also results in changes in fruit tissue morphology and reorganization of cell wall structure [79]. In addition, the Arabidopsis *P4H11* gene is involved in the regulation of ethylene biosynthesis. As an important plant hormone, ethylene has extensive effects on carbon metabolism during grain filling and maturation [80]. Therefore, the *P4H3* gene identified in the brown module may indirectly influence carbon partitioning between soluble sugars and starch by maintaining cell wall structural integrity and participating in hormone signaling regulation, thereby working synergistically with 60S ribosomal proteins to shape the awn-regulated seed carbon metabolic network.

## 5. Conclusions

This study systematically analyzed the effects of awns on thousand seed weight in *E. nutans* and revealed the underlying regulatory mechanisms at physiological and transcriptomic levels. Results confirmed that awn length in *E. nutans* has a positive effect on seed thousand seed weight. Awns are associated with and may contribute to increased thousand seed weight mainly through three pathways: first, as photosynthetic organs of the spike, they directly promote the synthesis and accumulation of storage substances such as soluble sugars and starch during seed development; second, they maintain cellular redox homeostasis during the critical period of grain filling and ensure normal metabolic processes by regulating the dynamic balance of antioxidant enzyme systems including SOD, CAT, and POD; third, they regulate seed development and substance accumulation at the molecular level by transcriptionally modulating the ascorbate and aldarate metabolism pathway and the expression of hub genes such as 60S ribosomal protein genes and prolyl 4-hydroxylase genes. This study systematically clarifies the regulatory mechanism of awns on thousand seed weight in *E. nutans* for the first time, providing an important theoretical basis for the genetic improvement of high-yield and high-quality germplasm in *Elymus* species.

## Figures and Tables

**Table 1:** 20 varieties of *E. nutans* germplasm.

Name	Origin	Altitude (m)	Longitude (E)	Latitude (N)
RRG-01	Ruoergai, Sichuan	3460	102°0′28″	33°0′30″
RRG-02	Ruoergai, Sichuan	3462	102°0′30″	33°0′44″
RRG-03	Ruoergai, Sichuan	3463	102°0′30″	33°0′44″
QL20-01	Qilian, Qinghai	3420	100°0′56″	37°0′58″
QL20-02	Qilian, Qinghai	3420	100°0′56″	37°0′58″
QL20-03	Qilian, Qinghai	3010	100°0′27″	38°0′4″
QL20-04	Qilian, Qinghai	2870	100°0′17″	38°0′8″
MQ20-01	Maqu, Gansu	3460	102°0′4″	33°0′60″
MQ20-02	Maqu, Gansu	3460	102°0′4″	33°0′60″
MQ20-03	Maqu, Gansu	3671	101°0′55″	33°0′52″
XH20-01	Xiahe, Gansu	2490	102°0′48″	35°0′13″
XH20-02	Xiahe, Gansu	2540	102°0′49″	35°0′13″
XH20-03	Xiahe, Gansu	2750	102°0′41″	35°0′13″
PI655186	Xiahe, Gansu	2830	102°29′25″	35°11′10″
PI628698	Tianzhu, Gansu	2860	102°08′29″	37°19′15″
LQ20-01	Luqu, Gansu	3130	102°0′27″	34°0′36″
LQ20-02	Luqu, Gansu	3140	102°0′27″	34°0′36″
LQ20-04	Luqu, Gansu	3090	102°0′33″	34°0′34″
LQ20-05	Luqu, Gansu	3030	102°0′39″	34°0′32″
LQ20-06	Luqu, Gansu	3010	102°0′40″	34°0′30″

**Table 2:** The primer information for qRT-PCR.

Gene Name	Forward Primer	Reverse Primer
*EVM0012103*	GCGGACCATCGACAGCTAAT	CTCCCTCATCCTCACCTCCA
*EVM0135189*	TTCTTCGGACCTGACCAAGC	CCGCGGTTATCAGGTCTACC
*EVM0109947*	ATGCATCGTCATCCTCCGTC	TGACCAGAGTCGATTTGGCC
*EVM0125627*	ATCGATGGCCAGCAGTTCAA	ATGTAGAAGCCCCTGTTGCC
*EVM0045997*	TTAAGCTGGGGGAATCTGCC	CGCTCATCCACGGACATCTT
*EVM0062311*	TTCTTCGTCGTACTGGAGCG	TGGCATCTTCCTTGGTCACC
*EVM0071133*	CTTTGCTCTGATCCTTGCGC	GCACCACATCACCCTCATGA
*EVM0104129*	GAGGGAGATCGGCTTCATCG	TTCTTGCTCTTCCCCTGCAG
*EVM0021504*	TCGGATGTTGAAGAAGGCGG	CAGGCCTCATGCTCCAGAAA
*EVM0012818*	GATCTGCCGGTTGCCATACT	CGCTCATCCACGGACATCTT
*EVM0009441*	GGTGACCAAGGAAGATGCCA	AGCTAGCGTGGGACTTGTTC
*EVM0021185*	GACCCCAAGGATCTGCAGAC	AACAACCTCCCCAAAGCCAA
*EnGAPDH*	CACCATGAAGTCAAGGTGAAGG	GCCTTGTCCTTGTCAGTGAA

**Table 3:** Annotation information of hub genes.

Gene Name	Swissport Annotation	Module Membership	Gene Significance
*EVM0045997*	60S ribosomal protein L26-1	0.9881	0.8547
*EVM0062311*	60S ribosomal protein L11	0.9918	0.9134
*EVM0076991*	60S ribosomal protein L37a-1	0.9910	0.9336
*EVM0021504*	Probable prolyl 4-hydroxylase 3	0.9869	0.8664
*EVM0012818*	60S ribosomal protein L26-1	0.9931	0.9172
*EVM0064391*	60S ribosomal protein L38	0.9858	0.8691
*EVM0009441*	60S ribosomal protein L11	0.9862	0.8891
*EVM0021185*	60S ribosomal protein L12	0.9907	0.8749

## Data Availability

The data that support the findings of this study are available from the corresponding authors upon reasonable request.

## Appendix A

**Table A1:** Statistical table of sequencing data.

Samples	Clean Reads	Clean Bases	GC Content	% ≥ Q30
WA-1	24,527,161	7,302,127,373	53.70%	93.88%
WA-2	22,539,586	6,704,744,393	53.42%	93.19%
WA-3	22,957,611	6,817,681,242	53.42%	93.48%
WB-1	24,147,758	7,160,373,075	51.21%	93.47%
WB-2	23,573,335	6,990,708,151	51.68%	93.42%
WB-3	24,077,592	7,120,050,875	51.43%	93.43%
WC-1	23,629,583	7,035,079,017	54.92%	92.87%
WC-2	22,595,236	6,729,527,894	54.70%	93.17%
WC-3	20,436,459	6,081,209,719	54.66%	91.32%
YA-1	22,751,871	6,757,146,109	53.80%	93.74%
YA-2	22,373,278	6,630,159,389	53.29%	94.13%
YA-3	22,510,169	6,696,660,805	52.66%	93.31%
YB-1	23,355,148	6,935,000,144	52.33%	93.41%
YB-2	23,274,367	6,892,712,743	52.46%	94.00%
YB-3	23,011,195	6,836,659,658	52.35%	93.35%
YC-1	21,747,115	6,466,279,054	52.77%	93.63%
YC-2	22,860,563	6,799,145,996	52.59%	93.59%
YC-3	22,203,379	6,584,041,627	52.23%	93.90%

## References

[1] Qi F., Xing P., Bao Y., Wang H., Li X. (2020). Advances in Genetic Studies of the Awn in Cereal Crops. Chin. Bull. Bot..

[2] Li L., Liu P., Zhang L., Zhang H., Jia J., Gao L. (2021). Awn Genes Mapping and Correlation Analysis for Agronomic Traits in Wheat. J. Plant Genet. Resour..

[3] Richardson A., Jones H., Bartlett M. (2024). Grass awns: Morphological diversity arising from developmental constraint. Curr. Opin. Plant Biol..

[4] Petersen K.B., Kellogg E.A. (2022). Diverse ecological functions and the convergent evolution of grass awns. Am. J. Bot..

[5] Elbaum R., Zaltzman L., Burgert I., Fratzl P. (2007). The role of wheat awns in the seed dispersal unit. Science.

[6] Li J., Jiang M., Liu T., Xie S., Zhang Y., Zhan R. (2024). Research Progress in Barley Awn. Barley Cereal Sci..

[7] Li X., Tang Y., Zhou C., Lv J. (2023). Contributions of glume and awn to photosynthesis, 14C assimilates and grain weight in wheat ears under drought stress. Heliyon.

[8] Maydup M.L., Antonietta M., Guiamet J.J., Graciano C., López J.R., Tambussi E.A. (2010). The contribution of ear photosynthesis to grain filling in bread wheat (*Triticum aestivum* L.). Field Crops Res..

[9] Rebetzke G.J., Bonnett D.G., Reynolds M.P. (2016). Awns reduce grain number to increase grain size and harvestable yield in irrigated and rainfed spring wheat. J. Exp. Bot..

[10] Olugbemi L.B., Bingham J., Austin R.B. (1976). Ear and flag leaf photosynthesis of awned and awnless *Triticum* species. Ann. Appl. Biol..

[11] Motzo R., Giunta F. (2002). Awnedness affects grain yield and kernel weight in near-isogenic lines of durum wheat. Aust. J. Agric. Res..

[12] Sanchez-Bragado R., Kim J.W., Rivera-Amado C., Molero G., Araus J.L., Savin R., Slafer G.A. (2020). Are awns truly relevant for wheat yields? A study of performance of awned/awnless isogenic lines and their response to source–sink manipulations. Field Crops Res..

[13] Sanchez-Bragado R., Molero G., Araus J.L., Slafer G.A. (2023). Awned versus awnless wheat spikes: Does it matter?. Trends Plant Sci..

[14] Afshari-Behbahanizadeh S., Akbari G.A., Shahbazi M., Sanjari S., Rizza F., Badeck F.W., Farahani L., Alahdadi I. (2024). Barley awn dimensions and barbs changes under terminal drought stress and its relation to grain yield and carbon isotope discrimination. Cereal Res. Commun..

[15] Hosseini S.M., Poustini K., Siddique K.H.M., Palta J.A. (2012). Photosynthesis of barley awns does not play a significant role in grain yield under terminal drought. Crop Pasture Sci..

[16] Grundbacher F. (1963). The physiological function of the cereal awn. Bot. Rev..

[17] AuBuchon-Elder T., Coneva V., Goad D.M., Jenkins L.M., Yu Y.Q., Allen D.K., Kellogg E.A. (2020). Sterile Spikelets Contribute to Yield in Sorghum and Related Grasses. Plant Cell.

[18] Gu B., Zhou T., Luo J., Liu H., Wang Y., Shangguan Y., Zhu J., Li Y., Sang T., Wang T. (2015). An-2 encodes a cytokinin synthesis enzyme that regulates awn length and grain production in rice. Mol. Plant.

[19] Aguirre M., Kiegle E., Leo G., Ezquer I. (2018). Carbohydrate reserves and seed development: An overview. Plant Reprod..

[20] Chen Y., Zhou S., Wang C., Li H., Huang D., Wang Z., Zhou D., Zhao L., Gong R., Pan Y. (2022). Analysis of Metabolite Differences in the Process of Grain Development of High-Quality Rice ‘Huanghuazhan’. Mol. Plant Breed.

[21] Zhao H., Wang J., Zhang Q., Zhao Q., Mei S., Liu X., Cheng F. (2015). Activities of Several Starch Synthesis Enzymes in Filling Grains for Rice Sugary Endosperm Mutant(Sug-11)and Its Relation to Starch Quality. Chin. J. Rice Sci..

[22] Zhang S., Ghatak A., Bazargani M., Kramml H., Zang F., Gao S., Ramsak Z., Gruden K., Varshney R., Jiang D. (2024). Cell-type proteomic and metabolomic resolution of earlyand late grain filling stages of wheat endosperm. Plant Biotechnol. J..

[23] Zhang K., Guo L., Cheng W., Liu B., Li W., Wang F., Xu C., Zhao X., Ding Z., Zhang K. (2020). SH1-dependent maize seed development and starch synthesis via modulating carbohydrate flow and osmotic potential balance. BMC Plant Biol..

[24] Liu J., Fan X., You M., Wang S., Zong R. (2016). Changes in sugar, pyruvic acid content and nitrate reductase activity of *Elymus sibiricus* reproductive branches during seed development. Acta Prataculturae Sin..

[25] Kumar A., Sharma A., Sharma R., Choudhary A., Srivastava P., Kaur H., Padhy A.K. (2022). Morpho-physiological evaluation of *Elymus semicostatus* (Nees ex Steud.) Melderis as potential donor for drought tolerance in wheat (*Triticum aestivum* L.). Genet. Resour. Crop Evol..

[26] Cox T.S., Bender M., Picone C., Tassel D.L.V., Holland J.B., Brummer E.C., Zoeller B.E., Paterson A.H., Jackson W. (2002). Breeding perennial grain crops. Crit. Rev. Plant Sci..

[27] Xiong Y., Yuan S., Xiong Y.L., Li L.Y., Peng J.H., Zhang J., Fan X., Jiang C.Z., Sha L.N., Wang Z.T. (2025). Analysis of allohexaploid wheatgrass genome reveals its Y haplome origin in Triticeae and high-altitude adaptation. Nat. Commun..

[28] Li T., Tang S.J., Li W., Zhang S.B., Wang J.L., Pan D.F., Lin Z.L., Ma X., Chang Y.N., Liu B. (2023). Genome evolution and initial breeding of the Triticeae grass *Leymus chinensis* dominating the Eurasian Steppe. Proc. Natl. Acad. Sci. USA.

[29] Miao J.M., Zhang X.Q., Chen S.Y., Ma X., Chen Z.H., Zhong J.C., Bai S.Q. (2011). Gliadin analysis of *Elymus nutans* Griseb. from the Qinghai-Tibetan Plateau and Xinjiang, China. Grassl. Sci..

[30] Qiu Y.S., Zheng Y.Y., Xie W.G. (2022). Advances in breeding of *Elymus nutans* in China. Chin. J. Grassl..

[31] Fu J.J., Miao Y.J., Shao L.H., Hu T.M., Yang P.Z. (2016). De novo transcriptome sequencing and gene expression profiling of *Elymus nutans* under cold stress. BMC Genom..

[32] Luo X., Yan L.J., Li D.X., You M.H., Zhang J.B., Ji X.F., Lei X., Bai S.Q., Chang D. (2024). Screening and evaluation of drought resistance of wild *Elymus nutans* germplasm resources. Acta Agrestia Sin..

[33] Qi J., Liu W.H., Hamblin A., Che M.M. (2022). Zinc and cadmium tolerance in different ecotypes of *Elymus nutans* from alpine grassland of Qinghai-Tibet Plateau. Commun. Soil Sci. Plant Anal..

[34] Long J.T., Dong M.J., Wang C.Q., Miao Y.J. (2023). Effects of drought and salt stress on seed germination and seedling growth of *Elymus nutans*. PeerJ.

[35] Su Z., Zha X.O.Z., Shi S.Y., Han Y.J., Gu T., Zha X.W.M., Qiang H.H., Wu J.X., Bao S.H.N., Miao Y.J. (2026). Effects of cutting period on forage yield and nutritional quality of *Elymus nutans* cv. Baqing. Crops.

[36] Tan X.Q., Huang Y.W., Xiong D.W., Lv K., Chen F.Q. (2020). The effect of *Elymus nutans* sowing density on soil reinforcement and slope stabilization properties of vegetation-concrete structures. Sci. Rep..

[37] Song J.C., Yu X.J., Wei K.T., Wang P.B., Tong Y.S., Li Z., He Y.L., Wang H.B. (2021). Effects of nitrogen application on forage production performance and nutritional quality of *Elymus nutans* in alpine region. Acta Agrestia Sin..

[38] Long J.T., Gao X.L., Miao Y.J. (2025). Effects of environmental factors on the phenotypic traits and seed element accumulation of wild *Elymus nutans* in Tibet. Sci. Rep..

[39] Knott D.R. (1986). Effect of genes for photoperiodism, semidwarfism, and awns on agronomic characters in a wheat cross. Crop Sci..

[40] Weyhrich R.A., Carver B.F., Martin B.C. (1995). Photosynthesis and water-use efficiency of awned and awnletted near-isogenic lines of hard red winter wheat. Crop Sci..

[41] Yang X.Y., Ma Z.H., Wei Q., Niu Z.P., Chen A.Q., Hu Z.C., Wang L.S. (2023). Research progress on awns of common wheat. Tillage Cultiv..

[42] Lin Z.L., Du W.H. (2026). Effect of Awns on Photosynthesis and Yield of Triticale Under Water Deficit Compared to Flag Leaves. Plants.

[43] Qiu Y.S. (2023). Physiological Basis of Awn Length Variation and Effect of Awns on Seed Yield in *Elymus nutans*. Master’s Thesis.

[44] Langfelder P., Horvath S. (2008). WGCNA: An R package for weighted correlation network analysis. BMC Bioinf..

[45] Liu B.W., Wang R.J., Gong J.J., Zhu T.Q., Long S., Guo H., Liu T.Y., Yang P.Z., Xu Y.F. (2023). Combined Cold and Drought Stress-Induced Response of Photosynthesis and Osmotic Adjustment in *Elymus nutans* Griseb. Agronomy.

[46] Adnan M., Morton G., Hadi S. (2011). Analysis of rpoS and bola Gene Expression Under Various Stress-Induced Environments in Planktonic and Biofilm Phase Using 2−ΔΔCT Method. Mol. Cell. Biochem..

[47] Ungaro A., Pech N., Martin J.-F., McCain R.J.S., Mévy P., Chappaz R., Gilles A. (2017). Challenges and advances for transcriptome assembly in non-model species. PLoS ONE.

[48] Duwayri M. (1984). Effect of flag leaf and awn removal on grain yield and yield components of wheat grown under dryland conditions. Field Crops Res..

[49] Ntakirutimana F., Xiao B.W., Xie W.G., Zhang J.C., Zhang Z.Y., Wang N., Yan J.J. (2019). Potential Effects of Awn Length Variation on Seed Yield and Components, Seed Dispersal and Germination Performance in Siberian Wildrye (*Elymus sibiricus* L.). Plants.

[50] Ntakirutimana F., Wan Y.Y., Liu W.H., Xie W.G. (2021). Contribution of awns to seed yield and seed shattering in Siberian Wildrye Grown under irrigated and rainfed environments. Agronomy.

[51] Baud S., Boutin J., Miquel M., Lepiniec L., Rochat C. (2002). An integrated overview of seed development in *Arabidopsis thaliana* ecotype WS. Plant Physiol. Biochem..

[52] Weber H., Borisjuk L., Wobus U. (2005). Molecular physiology of legume seed development. Annu. Rev. Plant Biol..

[53] Li X.J., Wang H.G., Li H.B., Zhang L.Y., Teng N.J., Lin Q.Q., Wang J., Kuang T.Y., Li Z.S., Li B. (2006). Awns play a dominant role in carbohydrate production during the grain-filling stages in wheat (*Triticum aestivum*). Physiol. Plant.

[54] Lehner A., Bailly C., Flechel B., Poels P., Côme D., Corbineau F. (2006). Changes in wheat seed germination ability, soluble carbohydrate and antioxidant enzyme activities in the embryo during the desiccation phase of maturation. J. Cereal Sci..

[55] Ait Hamnou R., Ben El Caid M., Hachrouni C., Dahou S. (2023). Germination enhancement, antioxidant enzyme activity, and metabolite changes in late Argania spinosa kernels under salinity. J. Arid Environ..

[56] Peng F., Weselake R. (2011). Gene coexpression clusters and putative regulatory elements underlying seed storage reserve accumulation in *Arabidopsis*. BMC Genom..

[57] Han J.G., Mao P.S., Niu Z.L., Sun R.C. (2000). Physiological and biochemical changes during seed development of *Elymus sibiricus*. Acta Agrestia Sin..

[58] Zheng Y.Y., Lin X.S., Xie W.G., Liu W.X. (2024). Full-length transcriptome and co-expression network analysis reveal molecular mechanisms of seed development in *Elymus sibiricus*. Seed Sci. Res..

[59] Bailly C. (2004). Active oxygen species and antioxidants in seed biology. Seed Sci. Res..

[60] Wei J., Xu C., Li K.X., He H.J., Xu Q.J. (2020). Progress on superoxide dismutase and plant stress resistance. Plant Physiol. J..

[61] Yuan M.D., Hou Z.X., Zhai M.P., Su Y. (2008). The Research Advances on Indole- 3- acetic acid (IAA) Catabolism Related Enzymes: IAA oxidase (IAAO), Peroxidase (POD). Chin. Agric. Sci. Bull..

[62] Liu Y.F., Wang W.W., Zu Y.X., Mei Y., Zheng J.Q., Wu Y.C., Guo J., Chen Z.B. (2019). Research Progress on the Effects of Catalase on Plant Stress Tolerance. Barley Cereal Sci..

[63] Sun C.H., Zhu J.K., Zhu Y., Cao J.X., Zhang J., Zhang Y.Q., Zhou H.J., Zhu Y.G., Ji Y.M., Ding R. (2024). Transcriptome analysis of the coexpression network of genes related to antioxidant characteristics after grain filling in purple rice. Sci. Rep..

[64] Zhang C.X., Feng B.H., Chen T.T., Zhang X.F., Tao L.X., Fu G.F. (2017). Sugars, antioxidant enzymes and IAA mediate salicylic acid to prevent rice spikelet degeneration caused by heat stress. Plant Growth Regul..

[65] Guo X.B., Tang Y.L., Sun X.F., Tang K.X. (2011). Metabolism regulation of vitamin C and E in higher plants. Plant Physiol. J..

[66] Arrigoni O., De Tullio M.C. (2002). Ascorbic acid: Much more than just an antioxidant. Biochim. Biophys. Acta Gen. Subj..

[67] Lv Y.Y., Zhang S.B., Wang J.S., Hu Y.S. (2016). Quantitative Proteomic Analysis of Wheat Seeds during Artificial Ageing and Priming Using the Isobaric Tandem Mass Tag Labeling. Int. J. Mol. Sci..

[68] Zhang S., Cao L.N., Sun X., Yu J.J., Xu X.Y., Chang R.H., Suo J.F., Liu G.J., Xu Z.R., Qu C.P. (2021). Genome-wide analysis of *UGDH* genes in *Populus trichocarpa* and responsiveness to nitrogen treatment. 3 Biotech.

[69] Thakur N., Flowerika S., Chaturvedi S., Tiwari S. (2023). Wheat derived glucuronokinase as a potential target for regulating ascorbic acid and phytic acid content with increased root length under drought and ABA stresses in *Arabidopsis thaliana*. Plant Sci..

[70] Linster C.L., Clarke S.G. (2008). L-Ascorbate biosynthesis in higher plants: The role of *VTC2*. Trends Plant Sci..

[71] Hasanuzzaman M., Bhuyan M.H.M.B., Anee T.I., Parvin K., Nahar K., Al Mahmud J., Fujita M. (2019). Regulation of Ascorbate-Glutathione Pathway in Mitigating Oxidative Damage in Plants under Abiotic Stress. Antioxidants.

[72] Zheng L., Zhou P.J., Pan Y.L., Li B.J., Shen R.F., Lan P. (2023). Proteomic profile of the germinating seeds reveals enhanced seedling growth in *Arabidopsis rpp1a* mutant. Plant Mol. Biol..

[73] Wang T., Chang Y.M., Zhao K., Dong Q., Yang J. (2022). Maize RNA 3′-terminal phosphate cyclase-like protein promotes 18S pre-rRNA cleavage and is important for kernel development. Plant Cell.

[74] Ma B., Liu H., Xiu Z.H., Yang H.H., Wang H.Q., Wang Y., Tang B.C. (2024). Defective kernel 58 encodes an Rrp15p domain-containing protein essential to ribosome biogenesis and seed development in maize. New Phytol..

[75] Li B.J., Zheng L., Wang R.A., Xue C.W., Shen R.F., Lan P. (2022). A proteomic analysis of *Arabidopsis* ribosomal phosphoprotein *P1A* mutant. J. Proteom..

[76] Tian S.J., Wu J.J., Liu Y., Huang X.R., Li F., Wang Z.D., Sun M.X. (2017). Ribosomal protein NtRPL17 interacts with kinesin-12 family protein NtKRP and functions in the regulation of embryo/seed size and radicle growth. J. Exp. Bot..

[77] Kojima H., Suzuki T., Kato T., Enomoto K., Sato S., Kato T., Tabata S., Saez-Vasquez J., Manuel E., Nakagawa T. (2007). Sugar-inducible expression of the nucleolin-1 gene of *Arabidopsis thaliana* and its role in ribosome synthesis, growth and development. Plant J..

[78] Kutyrieva-Nowak N., Leszczuk A., Denic D., Bellaidi S., Blazakis K., Gemeliari P., Lis M., Kalaitzis P., Zdunek A. (2024). In vivo and ex vivo study on cell wall components as part of the network in tomato fruit during the ripening process. Hortic. Res..

[79] Kutyrieva-Nowak N., Leszczuk A., Ezzat L., Kaloudas D., Zając A., Szymańska-Chargot M., Skrzypek T., Krokida A., Mekkaoui K., Lampropoulou E. (2024). The modified activity of prolyl 4 hydroxylases reveals the effect of arabinogalactan proteins on changes in the cell wall during the tomato ripening process. Front. Plant Sci..

[80] Bensassi K. (2014). Involvement of an Arabidopsis Prolyl 4 Hydroxylase 11 T-DNA Mutant in Ethylene Production. Master’s Thesis.

